# Exploration of wheat and pathogen transcriptomes during tan spot infection

**DOI:** 10.1186/s13104-018-3993-2

**Published:** 2018-12-19

**Authors:** Paula Moolhuijzen, Pao Theen See, Caroline S. Moffat

**Affiliations:** Centre for Crop Disease and Management, Curtin University, Perth, WA Australia

**Keywords:** *Pyrenophora tritici*-*repentis*, Transcriptome, ToxA, RNA-seq, Tan spot, *Triticum aestivum*, Wheat, Yellow spot

## Abstract

**Objectives:**

The fungus *Pyrenophora tritici*-*repentis* is the causal agent of tan spot, a major disease of wheat (*Triticum aestivum*). Here, we used RNA sequencing to generate transcriptional datasets for both the host and pathogen during infection and during in vitro pathogen growth stages.

**Data description:**

To capture gene expression during wheat infection with the *P. tritici*-*repentis* isolate M4, RNA datasets were generated for wheat inoculated with *P. tritici*-*repentis* (infection) and a mock (control) at 3 and 4 days post-infection, when scorable leaf disease symptoms manifest. The *P. tritici*-*repentis* isolate M4 was also RNA sequenced to capture gene expression in vitro at two different growth stages: 7-day old vegetative mycelia and 9-day old sporulating mycelia, to coincide with a latent growth stage and early sporulation respectively. In total, 6 RNA datasets are available to aid in the validation of predicted genes of *P. tritici*-*repentis* and wheat. The datasets generated offer an insight into the transcriptomic profile of the host–pathogen interaction and can be used to investigate the expression of a subset of transcripts or targeted genes prior to designing cost-intensive RNA sequencing experiments, that would be best further explored with replication and a time series analysis.

## Objective

The necrotrophic fungal pathogen *Pyrenophora tritici*-*repentis* causes tan spot disease of wheat (*Triticum aestivum*). Tan spot is an economically significant leaf disease, which has a major impact on the wheat industry worldwide. Here, we present exploratory RNA sequence data sets with the following aims: (1) to investigate *in planta* gene expression of both the host and pathogen during wheat tan spot infection by *P. tritici*-*repentis*, (2) to investigate in vitro *P. tritici*-*repentis* gene expression during vegetative and sporulating growth stages, and (3) to provide RNA sequencing for bioinformatics support of gene predictions in *P. tritici*-*repentis* [1] and wheat.

## Data description

In total, six RNA libraries were Illumina HiSeq sequenced to yield 24 and 25 million read pairs respectively for 3 and 4 days post-infection with *P. tritici*-*repentis*, 28 and 23 million read pairs respectively for 3 and 4 days post-inoculation of control wheat, and 23 and 26 million read pairs for 7-day old vegetative fungal mycelia and 9-day old sporulating mycelia respectively (Data file 1) (Table 1). The time points were chosen to maximise the appearance of early disease symptoms *in planta* and capture a latent growth and sporulating growth phase in vitro.

**Table 1:** Overview of data files/data sets

Label	Name data set	File types (file extension)	Data repository and identifier
Supplementary file 1	Methodology description	Word document (.docx)	10.6084/m9.figshare.7019843
Data file 1	Table of RNA sample collection and statistics	Spreadsheet (.xlsx)	10.6084/m9.figshare.7019843
Data file 2	Wheat gene expression 3 and 4 days post-infection	Spreadsheet (.xlsx)	10.6084/m9.figshare.7019843
Data file 3	*Pyrenophora tritici*-*repentis* gene expression *in planta* at days 3 and 4 days post-infection	Spreadsheet (.xlsx)	10.6084/m9.figshare.7019843
Data file 4	*Pyrenophora tritici*-*repentis* (isolate M4) gene expression in vitro of vegetative and sporulating mycelia	Spreadsheet (.xlsx)	10.6084/m9.figshare.7019843
Data set 5	Sample C1, 3 days post-control inoculation *in planta*	Fastq (fastq.gz)	European Nucleotide Archive (ENA) Run accession ERR2822756 (https://www.ebi.ac.uk/ena/data/view/ERR2822756)
Data set 6	Sample C2, 4 days post-control inoculation *in planta*	Fastq (fastq.gz)	European Nucleotide Archive (ENA) Run accession ERR2822757 (https://www.ebi.ac.uk/ena/data/view/ERR2822757)
Data set 7	Sample M1, 7-day in vitro vegetative mycelia	Fastq (fastq.gz)	European Nucleotide Archive (ENA) Run accession ERR2822758 (https://www.ebi.ac.uk/ena/data/view/ERR2822758)
Data set 8	Sample M2, 9-day in vitro sporulating mycelia	Fastq (fastq.gz)	European Nucleotide Archive (ENA) Run accession ERR2822759 (https://www.ebi.ac.uk/ena/data/view/ERR2822759)
Data set 9	Sample P1, 3 DPI *P. tritici*-*repentis* infection *in planta*	Fastq (fastq.gz)	European Nucleotide Archive (ENA) Run accession ERR2822760 (https://www.ebi.ac.uk/ena/data/view/ERR2822760)
Data set 10	Sample P2, 4 DPI *P. tritici*-*repentis* infection *in planta*	Fastq (fastq.gz)	European Nucleotide Archive (ENA) Run accession ERR2822761 (https://www.ebi.ac.uk/ena/data/view/ERR2822761)

To determine host gene expression during *P. tritici*-*repentis* infection, datasets from infected and non-infected leaf samples were individually aligned to the Chinese Spring wheat genome (IWGS V1.0) [2]. Over half of the reads for each dataset mapped to the wheat genome (Data file 1). A total of 33,449 genes (24%) of the 137,056 high-confidence wheat reference genes were detected in both the control and infected groups (Data file 2).

For *P. tritici*-*repentis* expression during host infection, datasets from 3 and 4 days post-infection were also individually aligned to the *P. tritici*-*repentis* genome of isolate M4 [1]. Only 0.4–0.6% of the sample reads mapped to the genome (Data file 1). A total of 9101 and 9824 transcripts were detected at 3 and 4 days post-infection respectively (Data file 3).

To profile *P. tritici*-*repentis* genes expressed at different mycelia growth stages, the in vitro vegetative and sporulating datasets were individually aligned to the M4 genome [1] with approximately half of the reads in concordant alignment (Data file 1). *A* total of 10,933 M4 transcripts were expressed in vitro and of these 8548 transcripts were found expressed in both vegetative and sporulating mycelia (Data file 4).

## Methodology

### Plant and fungal material

The fully extended leaves of the 2-week-old susceptible wheat (*Triticum aestivum*) variety Machete were inoculated with the *P. tritici*-*repentis* race 1 M4 isolate or a mock control solution [3]. Infected and control leaves were collected at 3 and 4 days post-inoculation (DPI). In vitro M4 samples of vegetative mycelia and sporulating mycelia grown on V8PDA agar [3] were harvested at 7 days and 9 days respectively. All samples were snap frozen in liquid nitrogen immediately after harvest, and stored at − 80 °C prior to RNA extraction.

### RNA extraction and sequencing

RNA was extracted using TRIzol Reagent (Thermo Fisher Scientific, USA), further purified using Zymo-Spin columns (Zymo Research, USA) as per the manufacturer’s guidelines prior to LiCl precipitation. RNA samples were pooled from 3 biological replicates. Isolated RNA was ribo-depleted and sequenced as un-stranded, 100 bp pair-end (PE) reads on an Illumina HiSeq2000 machine. A total of 30.6 Gb of raw sequence of 6 libraries was obtained. Further method details can be found in Supplementary file 1.

### Sequence analysis

Reads were quality checked with FASTQC [4] and trimmed using TrimmomaticPE V0.32 [5]. The trimmed reads were aligned to the *P. tritici*-*repentis* M4 reference genome (NCBI GenBank accession NQIK00000000.1) [1] and wheat Chinese Spring genome IWGS V1.0 [2] using Bowtie2/TopHat2 version 2.0.9 [6, 7]. Expression analysis was conducted with the Cufflinks package guided by the reference genes for M4 and high confidence genes in wheat [8].

## Limitations

The data sets generated were pooled from three biological RNA samples and therefore have no replicates for differential expression studies. The downloadable sequence data is stored raw and requires quality filtering before use.

## Abbreviations

DPI: days post-inoculation
RNA-seq: RNA sequencing
PE: paired-end
